# Mosquito Host Selection Varies Seasonally with Host Availability and Mosquito Density

**DOI:** 10.1371/journal.pntd.0001452

**Published:** 2011-12-20

**Authors:** Tara C. Thiemann, Sarah S. Wheeler, Christopher M. Barker, William K. Reisen

**Affiliations:** Center for Vectorborne Diseases, School of Veterinary Medicine, University of California Davis, Davis, California, United States of America; University of California Irvine, United States of America

## Abstract

Host selection by vector mosquitoes is a critical component of virus proliferation, particularly for viruses such as West Nile (WNV) that are transmitted enzootically to a variety of avian hosts, and tangentially to dead-end hosts such as humans. *Culex tarsalis* is a principal vector of WNV in rural areas of western North America. Based on previous work, *Cx. tarsalis* utilizes a variety of avian and mammalian hosts and tends to feed more frequently on mammals in the late summer than during the rest of the year. To further explore this and other temporal changes in host selection, bloodfed females were collected at a rural farmstead and heron nesting site in Northern California from May 2008 through May 2009, and bloodmeal hosts identified using either a microsphere-based array or by sequencing of the mitochondrial cytochrome c oxidase I (*COI*) gene. Host composition during summer was dominated by four species of nesting Ardeidae. In addition, the site was populated with various passerine species as well as domestic farm animals and humans. When present, *Cx. tarsalis* fed predominantly (>80%) upon the ardeids, with Black-crowned Night-Herons, a highly competent WNV host, the most prevalent summer host. As the ardeids fledged and left the area and mosquito abundance increased in late summer, *Cx. tarsalis* feeding shifted to include more mammals, primarily cattle, and a high diversity of avian species. In the winter, Yellow-billed Magpies and House Sparrows were the predominant hosts, and Yellow-billed Magpies and American Robins were fed upon more frequently than expected given their relative abundance. These data demonstrated that host selection was likely based both on host availability and differences in utilization, that the shift of bloodfeeding to include more mammalian hosts was likely the result of both host availability and increased mosquito abundance, and that WNV-competent hosts were fed upon by *Cx. tarsalis* throughout the year.

## Author Summary

West Nile virus (WNV) is transmitted from one vertebrate host to another by the bite of a mosquito. The virus is maintained primarily in birds, but can also be transmitted to mammals such as horses and humans which may suffer severe neurological disease. *Culex tarsalis* is a primary mosquito vector of WNV in the western United States. Because this mosquito will bite a variety of host species, understanding bloodfeeding patterns and host selection is important for understanding WNV transmission. In our study, the bloodfeeding patterns of *Cx. tarsalis* varied markedly throughout the year. During summer nesting herons were utilized almost exclusively; avian host diversity increased in the fall, when an increase in the proportion of bloodfeeding on mammals was also observed. Yellow-billed Magpies and House Sparrows were common hosts in the winter, when no mammalian bloodmeals were detected. Seasonal shifts corresponded to both changes in host availability and mosquito density; however, WNV-competent hosts were fed upon throughout the year. This work supports the role of *Cx. tarsalis* as a vector of WNV to both avian and mammalian hosts and provides insight into seasonal changes in host selection that may influence the seasonality of WNV transmission to equines and humans.

## Introduction

West Nile virus (WNV), a mosquito-borne zoonosis in the family *Flaviviridae*, is now endemic throughout the United States. Mosquitoes, primarily in the genus *Culex*, maintain the virus through transmission to a variety of avian host species that have varying competence for amplifying WNV. These mosquitoes also serve as bridge vectors to mammalian hosts, such as horses and humans, that may develop severe neurological disease [1]. Interestingly, most equine and human cases in California occur during late summer [2], [3], even though vector mosquitoes terminate diapause and begin actively bloodfeeding in midwinter [4]. Understanding the bloodfeeding patterns of vector mosquitoes, therefore, is important for appreciating how bite distribution on variably competent hosts can influence the maintenance and epidemic transmission dynamics of WNV and similar viruses.

*Culex tarsalis* is a highly competent vector of WNV [5], [6] found primarily in rural areas of the western United States [7]. Early bloodmeal identification studies from California and Colorado revealed that *Cx. tarsalis* is an opportunistic feeder, utilizing a variety of avian and mammalian species as hosts [8]–[11]. Recent studies have supported these findings, with >25 avian species and >5 mammalian species, including humans, identified from *Cx. tarsalis* bloodmeals [12], [13]. As a generalist feeder, *Cx. tarsalis* can serve as a maintenance, amplification, and bridge vector in the transmission of WNV, so discerning bloodfeeding patterns over time and space may help explain the variability seen in the dynamics of WNV transmission.

Several *Culex* species, including *Cx. tarsalis*, have exhibited a shift to increased mammal feeding during summer [10], [14], [15]. When Tempelis et al. [10] first described this phenomenon, it was hypothesized to be caused by a change in host availability, an increase in mosquito abundance leading to increased avian defensive behavior, and/or a physiological change in vector host preference. Subsequent cage studies supported the idea that avian defensive behavior increased with mosquito density and led to increased feeding on less defensive mammals [16], [17]. Increased mosquito abundance was likely the cause of a shift to mammalian feeding in *Culex nigripalpus* that could not be explained by host availability [15]. In urban *Culex pipiens*, a shift from primarily avian bloodfeeding to more mammalian bloodmeals was ascribed to a decrease in preferred host availability, as American Robins dispersed and then migrated after breeding. This shift in feeding was associated temporally with an increased frequency of human bloodmeals and an increased number of human cases of WNV [14].

The current study explored seasonal changes in the bloodfeeding patterns and host preferences of *Cx. tarsalis* in an effort to determine the impact on WNV transmission at a well-characterized farmstead study area north of Davis, CA. This farmstead served as a communal nesting site for herons and egrets that were competent hosts and frequently were infected with WNV [18]. Previously, an ardeid nesting colony near Tokyo was shown to be an amplification focus of Japanese encephalitis virus transmitted by *Culex tritaeniorhynchus* [19], [20], an ecological equivalent of *Cx. tarsalis*. In addition, our unique study area provided a natural experiment to explore the effects of a dramatic change in avifauna and the impact of marked seasonal changes in vector abundance associated with rice culture along the neighboring Sacramento River. It was hypothesized that 1) patterns of host selection, including any shift to mammal feeding, would be due to changes in host availability and/or mosquito abundance; 2) host preference would play a critical role in host selection within seasons; and 3) prevalent bloodfeeding on WNV-competent hosts would increase WNV activity as measured by infection within the vector population.

## Methods

### Study Site

**Figure 1:**
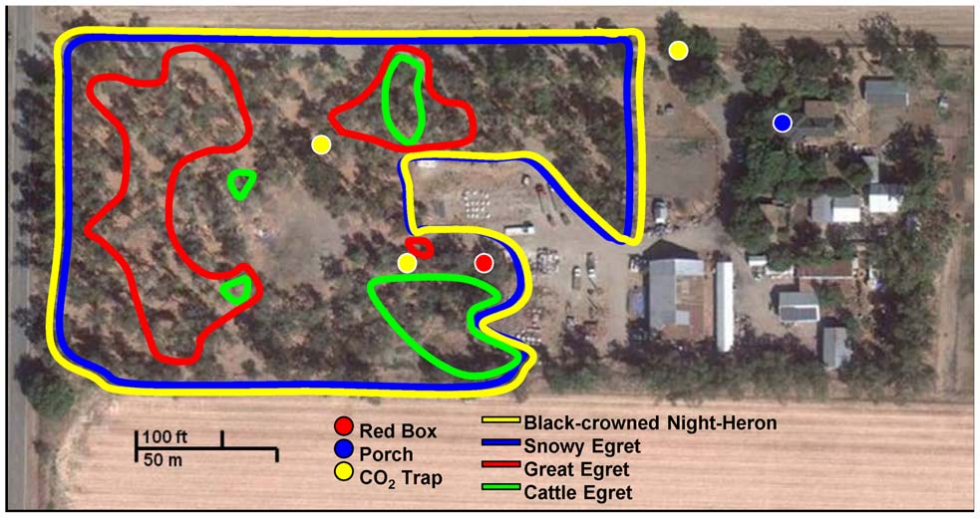
Map of study site. Map of the farmstead study site northeast of Davis, CA [38.6037°, −121.7094°] showing the locations of three dry-ice baited (CO_2_) traps and walk-in red box and porch resting collection sites. Approximate distributions of nesting herons and egrets were based on transect counts made in June 2008 and are delineated by colored outlines.

This study was conducted within a 4-hectare farmstead (Figure 1) located about 4 km NE of the town of Davis in Yolo County, CA [38.6037°, −121.7094°]. Much of the farmstead was covered by a stand of eucalyptus trees, which stood out from the surrounding pastures that dominated the landscape. The Yolo bypass of the Sacramento River, a large rice farming area, was located 3 km to the west. This and more proximate agricultural fields probably produced the majority of the mosquitoes in the area. The farmstead was inhabited by numerous wild avian species as well as humans and domestic animals including dogs, horses, cows, goats, chickens and geese. From mid-April to mid-September, roughly 10,000 birds in the family Ardeidae comprised of Black-crowned Night-Herons, Great Egrets, Snowy Egrets and Cattle Egrets communally nested in the farmstead's stand of eucalyptus trees. Nesting sites covered approximately 60% of the total land area of the farmstead, with the remaining land consisting of farmhouses, out buildings, and open-air animal enclosures. West Nile virus infection patterns in these ardeids were described previously [18]. Previous studies have implicated communal ardeid nesting areas as important sites for flavivirus amplification [19], [20]; however, comparable results have not been reported with WNV in North America.

### Mosquito Collection

To monitor vector abundance, mosquitoes were collected in dry-ice baited [21] CDC-style traps [22] operated without lights. Three traps (Figure 1) were operated one day per week from late afternoon to early morning from May through October of 2008 and January through June of 2009 at the heronry farmstead. Two additional traps were operated concurrently at the nearby Yolo Bypass Wildlife Area as a comparison site. Mosquitoes from each collection were anesthetized with triethylamine and enumerated by species. *Culex* species were pooled into groups of ≤50 females and tested for WNV using quantitative reverse transcription-polymerase chain reaction (qRT-PCR) [23].

From May 2008 until May 2009 resting female mosquitoes were aspirated once or twice weekly from a walk-in red box [24] and the covered porch of the farmhouse, spaced approximately 100 m apart (Figure 1). Bloodfed *Culex* mosquitoes were anesthetized and enumerated by species. Freshly fed, late fed, and half gravid *Cx. tarsalis* females [25] were stored individually at −80°C pending bloodmeal identification.

### Bloodmeal Identification and Diversity

Bloodmeal hosts were identified using methods previously described and evaluated [26]. Briefly, DNA was extracted from the mosquito abdomens using the DNeasy 96 Blood & Tissue Kit (Qiagen, Valencia, CA). A nested PCR was used to amplify the mitochondrial gene cytochrome c oxidase I (*COI*). Primers flanking *COI* first amplified the entire gene. The 658-bp ‘barcoding’ region of *COI* was then amplified using vertebrate-specific primers [27], [28]. Host DNA was first screened by a microsphere assay for *COI* developed for 15 frequently fed-upon hosts. Samples not identified by this assay were identified by sequencing this portion of *COI* and using the ‘Identify Specimen’ algorithm from Barcode of Life Data Systems (BOLD; www.boldsystems.org) [12], [29], [30]. The diversity of bloodmeal hosts was assessed biweekly in 2008 and monthly in 2009 using the Shannon diversity index (H′), where H′ = −Σ(*p_i_* ln *p_i_*) and *p_i_* was the proportion of bloodmeals for each host species. Bloodmeal host diversity was expressed as *e* ^H′^.

### Host Surveys

Nesting Black-crowned Night-Herons, Great Egrets, Snowy Egrets, and Cattle Egrets were censused in June 2008. Birds were counted along north-south transects spaced 15 m apart and divided into 15 m quadrants. These data were used to approximate the distribution of ardeid species within the farmstead in Figure 1. Occupied nests were counted and numbers were extrapolated to estimate adults and nestlings occupying the entire eucalyptus stand. Other avian and mammalian species were surveyed in August 2008 and March 2009. Counts were made within 1 hr of dawn from 6 points spaced equally around the farmstead to estimate the total number of available hosts roosting in the area. Domestic mammals on the farmstead were counted.

### Calculation of Host Selection

Host feeding selection for birds and mammals were calculated using the Manly selection ratio design I, a ratio in which utilized and available resources are estimated at the population level [31]. Statistical estimates were calculated using the adehabitat package in R [32]. The selection ratio (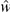 *_i_*) was calculated for nesting ardeids in June 2008 and for all available hosts in August 2008 and March 2009, where 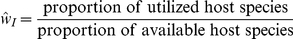
A selection ratio equal to 1 represented no host partiality, *i.e.* the frequency of mosquito feeding on a particular host was equal to that expected given the host's availability. Selection ratios >1 represented overutilization of hosts, whereas those <1 represented underutilization. Host species identified from bloodmeals, but not observed in host surveys, were assigned an available value of 1 individual for selection ratio calculations. Bloodmeals collected from 3 weeks before through 3 weeks after the host surveys were used to estimate the proportion of utilized hosts in early summer and winter when host populations were stable. Due to the transient state of host availability in late summer, when ardeid hosts were leaving the area and winter residents were arriving, bloodmeals from 10 days before through 10 days after the surveys were used for this calculation.

## Results

### Mosquito Abundance and WNV Activity

**Figure 2:**
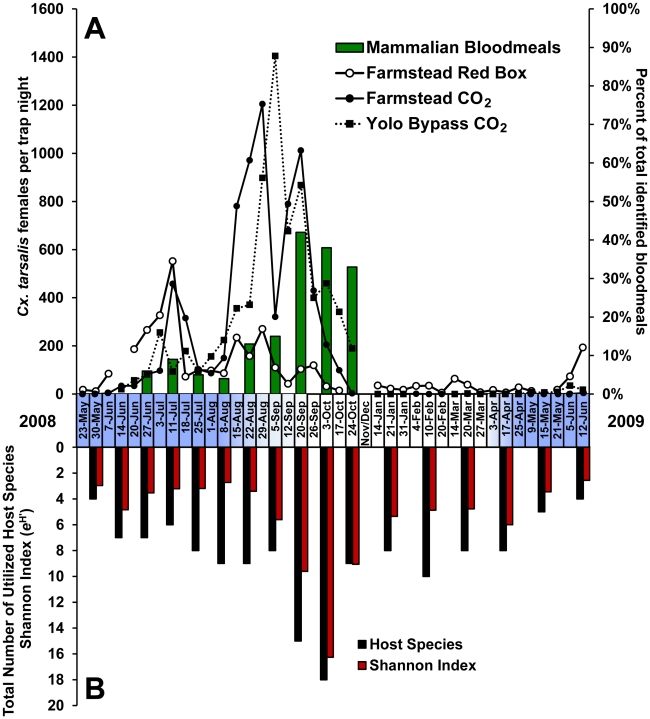
*Cx. tarsalis* abundance and host diversity. *Culex tarsalis* abundance and bloodmeal host diversity at a farmstead north of Davis, CA, from May 2008 through June 2009. Shading on the x-axis depicts the presence of nesting herons and egrets at the farmstead, with arrival beginning in April and departure in late August through September. A) Lines represent abundance of host-seeking females collected in CO_2_ traps at the farmstead and nearby Yolo Bypass Wildlife Area and resting females collected in the walk-in red box. Bars shows percent of total bloodmeals identified as mammalian hosts. B) Total number of utilized host species and Shannon diversity index (H′) expressed as *e* ^H′^. H′ increased both with increased number of hosts and the more even distribution of bloodmeals among host species.

Host-seeking *Cx. tarsalis* were abundant at traps from June through the end of October 2008 (Figure 2A). A small peak in abundance occurred in July, but host-seeking females were most abundant from the middle of August through the end of September. There was a drop in the number of females per trap night on September 5. A similar reduction occurred the following week at the neighboring Yolo Bypass Wildlife Area, and was attributed to mosquito adulticide applications near both sites. Generally, abundance patterns were similar at the farmstead and wildlife areas, reflecting mosquito production patterns associated with agriculture in this area. CO_2_ trap collections were extremely low (<2 females per trap night) during the winter months, but red box collections of reproductively active females remained relatively high, averaging >25 mosquitoes per week, thereby allowing bloodmeal identifications during this time.

Only 4 *Cx. tarsalis* mosquito pools tested positive for WNV from the farmstead in 2008, precluding statistical comparisons of WNV activity and *Cx. tarsalis* bloodfeeding patterns and, therefore, preventing the testing of the third hypothesis. Virus activity was also low for *Cx. tarsalis* in surrounding Yolo County during June through October 2008, with only 14 positive pools collected yielding an infection rate of 0.24 per 1,000 (95% confidence interval 0.14–0.40) for 1,429 pools comprising 57,632 females. As a point of interest, in August when the 4 WNV-positive pools were detected at the farmstead, Black-crowned Night-Herons were the most frequently fed upon host.

### Spatial Bias

When collected concurrently, there was no significant difference in host apportionment among females collected in the walk-in red box or from the farmhouse porch (χ^2^ = 4.83; d.f. = 7; p = 0.68). These results were congruent with a previous study that showed no significant differences between these collection sites [33], so the data from red box and porch collections were combined for all further analyses.

### Seasonal Bloodfeeding Patterns

**Figure 3:**
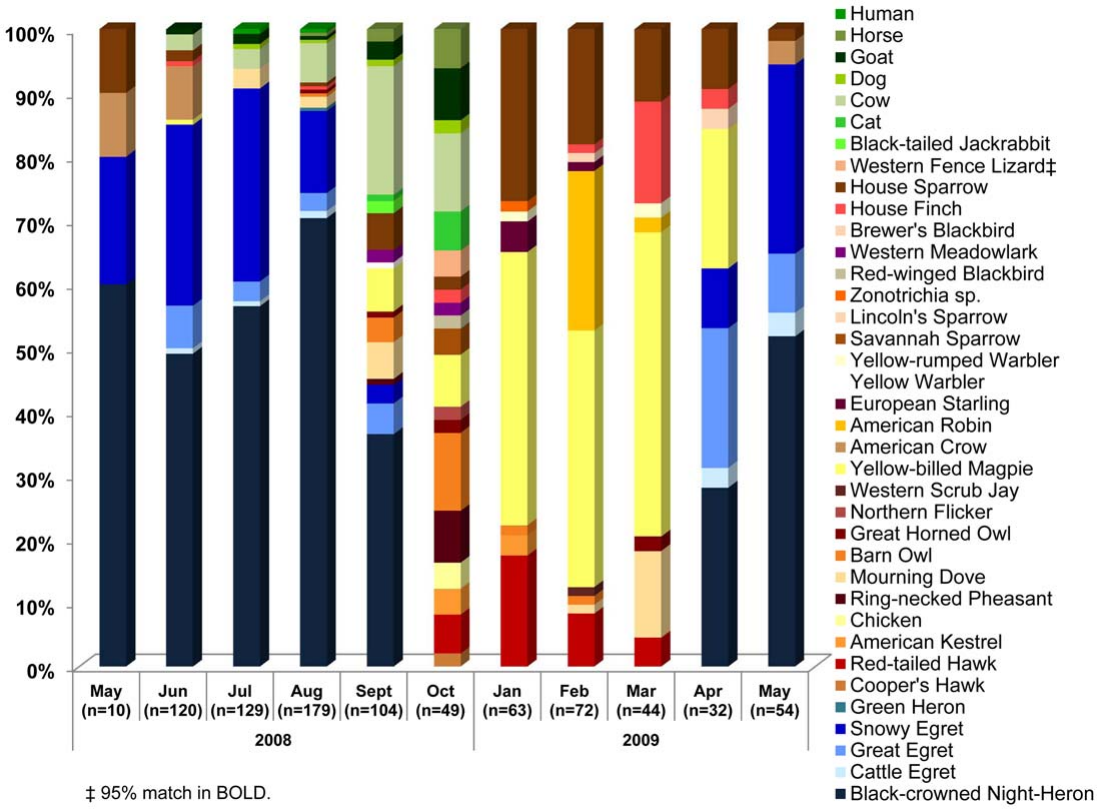
Percentage of bloodmeal hosts. Percentage of total identified bloodmeals from *Cx. tarsalis* collected resting at a farmstead north of Davis, CA from May 2008–May 2009. Herons and egrets are shown in shades of blue at the bottom of the graph, and mammals in shades of green at the top. Remaining colors represent other avian and reptilian species.

Blood-engorged *Cx. tarsalis* were collected in all months except November and December. 92% of the 925 tested bloodmeals were identified (80% by microsphere array and 12% by sequencing), yielding a total of 37 vertebrate host species identified from 851 bloodmeals (Figure 3). The 74 unidentified bloodmeals either did not produce a visible amplification product, likely due to digestion of the bloodmeal in half gravid and sub gravid individuals, no priming by the general vertebrate *COI* primers, or could not be conclusively identified to host species because of indistinguishable mixed sequence chromatograms or no host match in the database.

Only 3 (0.4%) of the bloodmeals were identified as mixed (i.e., consisting of blood from 2 host species) by the microsphere assay, with 1 each from cow/Mourning Dove, goat/Black-crowned Night-Heron, and Black-crowned Night-Heron/House Sparrow. Detection of mixed bloodmeals was limited because the microsphere assay only detected mixed bloodmeals when both species were among the 15 species for which we had a diagnostic probe. However, only 6 (2.1%) of the bloodmeals identified or confirmed by sequencing appeared to be mixed as indicated by multiple peaks in the chromatogram, so it seems that feeding on multiple hosts by *Cx. tarsalis* was rare, agreeing with previous reports based on trapping data [34], [35].

During May through August when nesting Ardeidae were present, >80% of the *Cx. tarsalis* fed on these birds, particularly Black-crowned Night-Herons and Snowy Egrets (Figure 3). During this period, relatively few (<10%) of the total bloodmeals came from mammals, and among these, cows were the most common host. Two human bloodmeals were identified in July and August, and these were the only human bloodmeals detected at this site throughout the year.

In late summer and autumn, bloodfeeding on mammalian hosts became more frequent, exceeding 30% of the total bloodmeals (Figure 2A). This shift to more prevalent mammalian feeding in September corresponded both to the departure of the Ardeidae and to peak *Cx. tarsalis* abundance host-seeking at dry ice baited traps (Figure 2A). Mosquito abundance decreased in October, but mammalian feeding, particularly on cows, goats and horses, remained frequent. In addition, a wide variety of avian species were utilized as hosts during this time (Figure 3). Both the total number of host species utilized and the Shannon diversity index peaked in October, indicating that bloodmeals were apportioned more evenly over a wider variety of host species (Figure 2B).

During winter, the diversity of utilized hosts again decreased (Figure 2B). Yellow-billed Magpies and House Sparrows were the predominant bloodmeal hosts (>60%), followed by American Robins, Red-tailed Hawks and House Finches. In the spring of 2009, when the ardeids returned, these birds again became the predominant hosts of *Cx. tarsalis*. Great Egrets were the first of these species to return to their nests in the spring, accounting for the high percentage of bloodmeals from this species in April (Figure 3).

### Host Selection Ratios

Within seasons, host selection was not equitable. Considering only the Ardeidae hosts in early summer of 2008, Snowy Egrets were fed upon significantly more than expected based on their abundance, whereas Cattle Egrets were utilized significantly less than expected (Table 1). Black-crowned Night-Herons were the most frequently fed-upon ardeid host, but they, like Great Egrets, were neither over nor under-utilized by *Cx. tarsalis* based on their estimated abundance.

**Table 1:** Host selection ratios (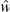 *_i_*) of *Cx. tarsalis* for four Ardeidae species during their communal nesting period, June 2008.

Host Species	Bloodmeals (%)	Available (%)	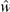 *_i_* (SE)
Black-crowned Night-Heron, *Nycticorax nycticorax*	126 (59)	6912 (64)	0.92 (0.05)
Snowy Egret, *Egretta thula*	73 (34)	2688 (25)	1.37 (0.13)*
Great Egret,2 *Ardea alba*	12 (6)	648 (6)	0.94 (0.27)
Cattle Egret, *Bubulcus ibis*	2 (1)	528 (5)	0.19 (0.14)*

*Statistically significant non-random host selection (p<0.05).

(SE) Standard Error.

Host availability varied between late summer 2008 and winter 2009, due to changes in avian nesting behavior and migration patterns. Twelve host species were available only in late summer, 10 hosts only during winter, and 17 during both time periods (Table 2). In late summer, Black-crowned Night-Herons and cows were fed upon more frequently than expected based on their abundance, whereas Snowy Egrets, Cattle Egrets and House Sparrows were fed upon less frequently than expected. Standard errors, and thus significance, could not be determined for hosts that were not utilized, but it is interesting that several host species including European Starlings, Yellow-billed Magpies and humans were not detected in *Cx. tarsalis* bloodmeals, even though they were available during this 20-day period and were fed upon elsewhere.

**Table 2:** Host selection ratios (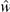 *_i_*) of *Cx. tarsalis* following the completion of nesting by the Ardeidae during host census periods in late summer 2008 and winter 2009.

	Late Summer			Winter		
Host Species	Bloodmeals # (%)	Available # (%)	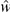 *_i_* (SE)	Bloodmeals # (%)	Available # (%)	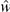 *_i_* (SE)
**Avian**						
Black-crowned Night-Heron, *Nycticorax nycticorax*	116 (69)	1728 (52)	1.32 (0.07)*			
Snowy Egret, *Egretta thula*	11 (7)	672 (20)	0.32 (0.1)*			
Mourning Dove, *Zenaida macroura*	7 (4)	2 (<1)	69.00 (55.05)	7 (6)	12 (1)	4.25 (1.98)
Great Egret, *Ardea alba*	7 (4)	162 (5)	0.85 (0.32)			
House Sparrow, *Passer domesticus*	2 (1)	300 (9)	0.13 (0.09)*	18 (16)	300 (36)	0.44 (0.10)*
Barn Owl, *Tyto alba*	1 (<1)	0 (<1)‡	19.71 (27.84)	1 (1)	0 (<1)‡	7.29 (10.29)
Great Horned Owl, *Bubo virginianus*	1 (<1)	0 (<1)‡	19.71 (27.84)	1 (1)	0 (<1)‡	7.29 (10.29)
House Finch, *Carpodacus mexicanus*	1 (<1)	20 (<1)	0.99 (1.01)	8 (7)	50 (6)	1.17 (0.43)
Cattle Egret, *Bubulcus ibis*	1 (<1)	132 (4)	0.15 (0.15)*			
European Starling, *Sturnus vulgaris*	0	150 (5)	0	1 (1)	150 (18)	0.05 (0.05)*
Turkey Vulture, *Cathartes aura*	0	15 (<1)	0			
Western Kingbird, *Tyrannus verticalis*	0	15 (<1)	0			
Yellow-billed Magpie, *Pica nuttalli*	0	10 (<1)	0	50 (43)	16 (2)	22.79 (6.15)*
American Crow, *Corvus brachyrhynchos*	0	5 (<1)	0	0	20 (2)	0
Brewer's Blackbird, *Euphagus cyanocephalus*	0	5 (<1)	0	0	75 (9)	0
Red-shouldered Hawk, *Buteo lineatus*	0	3 (<1)	0	0	1 (<1)	0
Red-tailed Hawk, *Buteo jamaicensis*	0	3 (<1)	0	8 (7)	1 (<1)	58.34 (61.61)
Anna's Hummingbird, *Calypte anna*	0	2 (<1)	0			
Domestic Goose, *Anser cygnoides*	0	2 (<1)	0			
Nuttall's Woodpecker, *Picoides nuttallii*	0	2 (<1)	0	0	2 (<1)	0
Black Pheobe, *Sayornis nigricans*	0	1 (<1)	0	0	1 (<1)	0
Chicken, *Gallus gallus*	0	1 (<1)	0			
Muscovi Duck, *Cairina moschata*	0	1 (<1)	0			
American Robin, *Turdus migratorius*				19 (16)	5 (<1)	27.71 (13.66)*
Western Scrub-Jay, *Aphelocoma californica*				1 (1)	0 (<1)‡	7.29 (10.29)
Lincoln's Sparrow, *Melospiza lincolnii*				1 (1)	0 (<1)‡	7.29 (10.29)
Yellow-rumped Warbler, *Dendroica coronata*				1 (1)	150 (18)	0.05 (0.05)*
Red-winged Blackbird, *Agelaius phoeniceus*				0	25 (3)	0
White-crowned Sparrow, *Zonotrichia leucophrys*				0	10 (1)	0
Northern Mockingbird, *Mimus polyglottos*				0	5 (<1)	0
Mallard, *Anas platyrhynchos*				0	2 (<1)	0
Orange-crowned Warbler, *Vermivora celata*				0	1 (<1)	0
Ruby-crowned Kinglet, *Regulus calendula*				0	1 (<1)	0
**Total Avian**	**147 (88)**	**3233 (98)**	**0.89**	**116 (100)**	**831 (98)**	**1.02**
**Mammalian**						
Cow, *Bos taurus*	16 (10)	6 (<1)	52.57 (24.82)*	0	2 (<1)	0
Goat, *Capra hircus*	2 (1)	53 (2)	0.74 (0.53)	0	10 (1)	0
Dog, *Canis familiaris*	2 (1)	4 (<1)	9.86 (8.5)	0	4 (<1)	0
Horse, *Equus caballus*	1 (<1)	3 (<1)	6.57 (7.57)	0	3 (<1)	0
Human, *Homo sapiens*	0	10 (<1)	0			
Cat, *Felis catus*	0	3 (<1)	0			
**Total Mammalian**	**21 (12)**	**79 (2)**	**5.24**	**0 (0)**	**15 (2)**	**0**

*Statistically significant non-random host selection (p<0.05).

**‡:** Species not observed during host survey. Given lowest available proportion for selection ratio calculation.

(SE) Standard Error.

At the time of the winter host survey, bloodmeals derived from Yellow-billed Magpies were the most prevalent (43%), followed by American Robins (16%) and House Sparrows (16%) (Table 2). Both magpies and robins were fed upon more frequently than expected given their low abundance at the farmstead, but, with an estimated population of ∼300 individuals, House Sparrows were under-utilized as a host. European Starlings and Yellow-rumped Warblers were also fed upon significantly less than expected from their high abundance. Contrary to the late summer, cows were not fed upon for *Cx. tarsalis* during the winter (Table 2). This was unexpected, because domestic mammals were present in corrals near our resting box during winter; however, mammalian bloodmeals were not detected from January through May 2009 (Figures 2A,3).

## Discussion

*Culex tarsalis* abundance and bloodfeeding patterns varied seasonally at a farmstead north of Davis, CA. Although there was little WNV activity in Yolo County during 2008–2009, it is interesting to note that WNV-competent hosts were fed upon commonly throughout the year. In fact, Black-crowned Night-Heron nestlings were experimentally found to be competent hosts for WNV [36] and previous studies at the heronry detected nestlings with serum viremia titers >8 log_10_ plaque forming units (PFU) of WNV/mL [18]. Four WNV-positive mosquito pools were detected at the farmstead in August 2008, when bloodmeals were detected from WNV-competent hosts as well as humans and horses. This highlights the potential for *Cx. tarsalis* to serve as a bridge vector for WNV, particularly in years with greater WNV activity.

### General Feeding Patterns

Herons and egrets were the most abundant and frequently utilized hosts during their nesting period from May through August. Mammalian bloodmeals increased in the late summer and fall after the ardeids left the study area, and passerine species, namely Yellow-billed Magpies, House Sparrows, and American Robins, were the most commonly fed upon hosts during the winter months. Host species utilization was related to both host abundance and selection by *Cx. tarsalis*. For example, Black-crowned Night-Herons were the most abundant (64%) and most frequently fed-upon (59%) host in early summer, whereas 43% of the winter bloodmeals were derived from an estimated population of only 16 resident Yellow-billed Magpies.

Snowy Egrets were over-utilized in June and under-utilized in August, while Black-crowned Night-Herons were over-utilized in August but fed upon as predicted in June. This discrepancy in host selection may have resulted from changes in mosquito abundance and host defensive behavior. Snowy Egrets are highly defensive toward mosquitoes [37], so it was unexpected that these birds were over-utilized in June, even when non-defensive Black-crowned Night-Herons [37] were readily available. However, mosquito host-seeking abundance was low in June so host defensive behavior may not have played a role host selection. In contrast, during August, when *Cx. tarsalis* abundance was at its peak, Snowy Egrets and other defensive hosts likely increased their anti-mosquito behaviors [16], [17], leading to an over-utilization of less defensive hosts such as Black-crowned Night-Herons and cows.

### Temporal Feeding Shifts

As expected from previous studies [7], [10], [38], *Cx. tarsalis* fed most frequently on mammals during late summer and fall. Mammalian bloodmeals were first detected in June when mosquito abundance began to increase. The shift to more frequent mammalian feeding in September corresponded to both a change in host availability, with adult and fledgling herons and egrets leaving the area, and to a marked increase in mosquito abundance. Interestingly, a short term depression in host-seeking abundance at this time due to mosquito control operations (Figure 2) did not appear to alter the increasing frequency of mammalian blood meals. Mammalian feeding remained frequent in October as mosquito abundance decreased but, mammalian bloodmeals were not detected during winter when mosquito abundance was at its lowest. Given these results, it seems likely that the increased mammalian feeding by *Cx. tarsalis* in our study resulted from the high density of vectors per available host. The impact of genetic, physiological, or other changes within the mosquito population were not explored, but may have contributed to this host shift. Humans were fed upon infrequently throughout (even when samples were taken from the house porch), undoubtedly reflecting their limited availability after sunset and/or their avoidance by host-seeking females. Comparable low frequency of human feeding was reported when *Cx. tarsalis* were collected at a county park on Monday morning after elevated evening use during the previous weekend [10].

In addition to increased mammalian feeding, *Cx. tarsalis* fed more equitably on a diverse range of hosts when vector density was highest. Both the number of utilized hosts and the Shannon diversity index was highest in October when the most frequently fed-upon host, Barn Owl, comprised only 12% of the total bloodmeals. During summer and winter, Shannon indices were low due to both a lower number of utilized hosts and disproportionate feeding on over-selected hosts such as Black-crowned Night-Herons and Yellow-billed Magpies, respectively.

### Underutilized Hosts

In general, small birds were under-utilized by *Cx. tarsalis*, agreeing with the low numbers detected in previous bloodmeal identification studies [12], [13] and their comparatively low WNV infection rates detected during serosurveys [39], [40]. WNV-competent House Sparrows were the most frequently fed upon of the smaller bird species at the farmstead, but they, too, were significantly under-utilized by *Cx. tarsalis*. Similarly, House Sparrows were under-utilized by both *Cx. pipiens* and *Cx. restuans* in Chicago, Illinois, but high abundance resulted in their comprising >15% of the total bloodmeals from these *Culex* species [41]. Though small in size, highly competent House Finches were not under-utilized in the current study, so, similar to Chicago [41], this host may be particularly important in WNV amplification even with lower total abundance.

Given the high number of WNV positives reported from dead bird surveys, it is notable that few *Culex* bloodmeals have been identified previously from American Crows [12], [13], [41], [42]. Supporting this trend, American Crows were under-utilized in the current study, with no bloodmeals detected in either the late summer or winter when American Crows were observed at the study site. However, during these time periods, crows foraged at the heronry during the morning hours but spent the night in communal roosts away from the farmstead. Therefore, although crows were included in the post-dawn host surveys, they were likely not available when the majority of *Cx. tarsalis* were host seeking during early evening [43]. During the late spring and early summer when American Crows were nesting at this and other sites, these birds were fed upon by *Cx. tarsalis* (Figure 3) as well as *Cx. pipiens* (unpublished data). In support of this scenario, Yellow-Billed Magpies, a similar corvid species, were over-selected during winter when a small group roosted at the farmstead.

### Summary

*Culex tarsalis* fed upon a wide assortment of birds and mammals, and the pattern of host utilization varied markedly throughout the year. Temporal changes in bloodfeeding patterns were associated with changes in host availability, increases in mosquito abundance during late summer, and *Cx. tarsalis* host preferences. The most abundant hosts at this study area, Black-crowned Night-Herons and Snowy Egrets, were the most frequent bloodmeal hosts in the summer, while Yellow-billed Magpies, a relatively rare host at the site, were the predominant winter host. We conclude that the late summer shift to more frequent mammalian feeding likely resulted from a combination of changing host species composition and increased host-seeking density. Despite these changing patterns, *Cx. tarsalis* fed frequently on WNV-competent hosts throughout the year and on mammals in the late summer and fall, indicating that *Cx. tarsalis* may serve as both a maintenance and a bridge vector of WNV.
